# Hierarchy of Factors Affecting the Condition and Development of Sports and Recreation Infrastructure—Impact on the Recreational Activity and Health of the Residents of a City (Poznan Case Study)

**DOI:** 10.3390/ijerph16040556

**Published:** 2019-02-14

**Authors:** Ewa Kruszyńska, Joanna Poczta

**Affiliations:** 1 The University of Szczecin, 70-453 Szczecin, Poland; ewa.kruszynska@usz.edu.pl; 2 Poznan University of Physical Education, 61-871 Poznan, Poland

**Keywords:** sport, recreation, sports infrastructure, health, physical activity, the Anderson–Darling test

## Abstract

The aim of the article is to identify which groups of factors (economic, social, and spatial) significantly determine the condition and development of the sports and recreation infrastructure of the city of Poznan and shape the needs and expectations of its residents. Standardized interviews among 39 service providers and 1159 service recipients made it possible to collect primary data on the presentation of the pace and directions of changes taking place in the sports and recreational facilities of the city, paying special attention to identifying and prioritizing factors determining this development. In order to establish the hierarchy of factors analyzed in the paper and operating within the same research problem for both groups of respondents (service recipients and service providers), the Anderson-Darling test was used. The test results were referenced to the already existing “*Sportowy Poznan*” (“Sports Poznan”) program. The assessment made by service recipients shows that the factor having the strongest limiting effect on the use of sports and recreation services is the economic factor. In the assessment made by the respondents using their services, the efforts of city authorities to make Poznan sports clubs operate in a professional manner gained the lowest score. The results of the Anderson–Darling test show that the social factor of preparing infrastructure for the residents of the city of Poznan was the most important for the respondents, obtaining a test value of 0.886.

## 1. Introduction

The issue of determining the position and importance of sports and recreation in the development of cities and regions is unquestionable. An example of such reasoning is the concept of the city as an “entertainment machine” created by T.N. Clark [1]. The author maintains that aesthetic factors currently prevail in the development of cities, which allows for the creation of appropriate conditions for sports and recreation. In Clark’s concept, sports and recreation are important elements of urban development strategies, including those traditionally dominated by industry. As an example, supporting his concept, Clark gives the case of Chicago, where the number of employees in the broadly understood sports and recreation (entertainment) sector has long exceeded the number of residents employed in the industry [1]. According to C.C. Williams [2], researchers who were among the first to deal with the economic benefits for local authorities resulting from sports activities were W.A. Shaffer and L.S. Davidson [3]. In 1984, they published a paper regarding the income of Atlanta authorities, due to the existence of the Atlanta Falcons, an American football team, in the city [3]. A particularly large number of studies on the role of sport in the development of the urban economy have been based on analyses carried out in Indianapolis (e.g., Kotler et al., Rosentraub et al.) [4,5]. The success of the strategy based on city authorities’ active support of services directly and indirectly connected with sport could be proved by studies carried out by M.S. Rosentraub et al. The researchers found that during a period of 15 years (1977–1989), the costs incurred for this purpose in Indianapolis had a 64% return rate, and employment increased by as much as 32.9%. As a result of this city’s successes, the development strategies used by Indianapolis (related to sport) were adopted by the municipalities of Cleveland, Jacksonville, Memphis, and Charlotte, as well as the British city of Sheffield [5]. Initially, the issue of the role of sports and recreation in the activation of the urban economy was mainly dealt with in the United States (e.g., Baltimore, Boston), and only slightly later in the UK (e.g., Glasgow, Bradford, Edinburgh), France (e.g., Lyon), Spain (e.g., Valencia), and other European countries. For more than a dozen years, the awareness of the importance of sports and recreation for urban development has become increasingly common [2]. It is not surprising that the question of the role of sports and recreation in the socio-economic activation of cities has also been raised in Poland and is increasingly the subject of detailed research. The broadly understood development of sports and recreation in cities may have an impact on: (1) Improving the quality of their residents’ lives (social aspect), (2) activating the local economy (economic aspect), (3) enriching the image of the city (psychological aspect), and (4) valorization of urban space (cultural and spatial aspect). In addition, the “Strategy for Sports Development in Poland until 2015” [6], which was adopted by the Council of Ministers on 23 January 2007, identified the actions necessary to achieve the level of physical education and sport corresponding to the standards of developed European Union countries in the perspective of the year 2015. This strategy distinguishes three priorities of actions: (1) Popularizing sport for all, (2) increasing the level of sports results, and (3) developing sports and recreation infrastructure. The necessity to create such strategies is a consequence of both fast-moving changes in the environment, as well as the intensification of globalization processes and competition among cities (for tourists, investments, funds, etc.) [6]. However, the types of sports and recreation infrastructure that exist in cities are highly diverse in terms of scale and urban and architectural solutions, from complex multifunctional sports and recreation centers, such as modern urban stadiums, to individual sports and recreation facilities (size and character are determined here by the types of sports and recreation disciplines involved, including the necessary background). In the current solutions, there is a tendency to equip cities with infrastructure that satisfies the maximum, and diverse number, of users and spectators. It is a phenomenon of the commercialization of the city space for sports and recreation.

Investments in sports and recreation infrastructure allow for the development of sport [7]. Among the sports and recreation facilities within the sports and recreation infrastructure, many different types can be distinguished: Marinas (harbors), golf courses, ice rinks, pitches, swimming pools, climbing walls, leisure centers, ski lifts, tennis courts, regatta tracks, bowling equipment, and more [7]. In Polish literature, there is the concept of material resources in physical culture, which can be treated as a synonym of the term “sports and recreation infrastructure,” for which Ryba includes financial resources, sports and recreation facilities, and sports equipment. Ryba defines sports and recreation facilities as open and volumetric spaces functionally connected with particular types of sport [8].

The growing number of business entities in sports and recreation, as well as the relations between them, both non-commercial and, increasingly often, commercial, reinforces the statement that Poland faces the process of the creation of a new model of sports and recreation services on the market. In Western European countries and in the United States, the sports market was defined as early as in the 1970s, and nowadays, e.g., in the USA, it ranks 11th in terms of turnover ($152 billion) among all industry markets [9]. A review of the subject literature proves that the researchers repeatedly attempted to build models of the sports and recreation market in order to determine its framework and subjective or objective structure. The most well-known models include:

- The sports and recreation market model developed by B. Pitts, L. Fielding, and L. Miller, who selected three large segments of this market: Entities dealing with organizing sport (sports clubs, recreation centres), entities producing material sports goods and representatives of units supporting sport (e.g., trainers, medical facilities, organizing committees), and entities that deal with sport promotion (sponsors, licensees, sports media, etc.) [10].
- A simplified model of the sports and recreation market developed by M. Shank, who assumes that the market is formed by two groups of entities: Recipients and providers, as well as sports products that are the subject of exchange [11].
- The model of the sports and recreation market proposed by B. Mullin, S. Hardy, and W. Sutton, where the sports and recreation market is depicted by means of four categories of entities or activities related to sport: Organizers of sports events with a high level of attractiveness, attracting a large audience; producers and distributors of tangible sports goods and government non-profit organizations; entities organizing sports life at the school and company level; and finally, supporting entities, such as sports associations, sponsors, and sport activists [12].

T. Slack et al., believes that an entity that acts directly for the benefit of sport or directly supports sport is any entity that operates on the sports market and treats it as its core area of activity (core business). In the group of providers, three types of entities are distinguished: (1) Sports organizations, e.g., sports clubs and other physical culture organizations, such as sports schools, sports associations, sports and recreation centers, etc.; (2) producers and distributors of sports goods (enterprises that, acting in a commercial manner, directly support sport and provide footwear, clothing, and sports equipment and accessories to final recipients; (3) service entities providing their services to both final recipients and institutional customers (including rental of sports equipment, repair points, sports tourism offices, sport marketing agencies, and managers and agents representing the interests of clubs or individual athletes) [13]. Thus, an urgent social task is to develop and disseminate methods and means of the rational use of sports and recreation infrastructure in order to increase the physical activity of society. These projects should be accompanied by the development of proper sports and recreation infrastructure, but this is a problem not so much of state policy as it is of the initiatives of the authorities of particular cities, specific associations, companies, and private entrepreneurs.

The presented studies are innovative and do not have sufficient support in the literature on the subject. Research on the participation of Poles in sports and recreational activities was published by the Central Statistical Office in 2013 [14]. Later in the article, the authors will refer to the results of these studies. In 2015, the Ministry of Sport and Tourism in Poland published the *Diagnosis of Social Requirements for Infrastructure, Sports, and Recreation* [15]. It presents the most popular sports practiced by Poles and which sports objects are most frequently used. Another document prepared in 2016 by Kostrzewska in the Ministry of Sport and Tourism in Poland is the overview of spatial solutions and activation programs in selected countries entitled *Sport for All in the City’s Space* [16]. In 2017, the same ministry issued a publication entitled *Best Practices in Sport Objects Managing* by Polanowski and Brach [17]. All these documents and research reports related to the preferences and needs of Poles in the field of sports and recreational activities, the demand for sports and recreation, and barriers that limit this activity. They contain practical guidelines for leading such objects for service providers. However, nobody has conducted detailed research for individual cities in Poland. Nobody has studied the factors affecting the condition and development of sports and recreation infrastructure or its impact on the sports activity of the residents not only of the city of Poznań, but in general.

Sports and recreation are important areas of activity for the city of Poznan. Each year, more than 10% of Poznan’s budget is spent on physical culture, which is a level comparable to that of the European Union average [18]. Poznan’s expenditures allocated to physical culture belong to the highest in the country and account for PLN 410 million, which is 12.3% of the annual budget of the city. Only Warsaw, which also allocates PLN 410 million a year for this purpose, can compete with Poznan, but this amount is only 3.3% of the capital’s budget [19]. Poznan undoubtedly strives to be a city that “puts on sport.” In the resolution of the City Council adopted in 2010, “Strategy for the City of Poznan until 2030,” one of the strategic goals is to increase the importance of the city as a center of sport. Poznan has over 500,000 residents [19]. The sports and recreation infrastructure are very complex, because it consists of various elements, from huge recreational complexes to modern large-area clubs to intimate places associated with the chosen form of recreation. The diverse offer of sports and recreation facilities proposed by the city for its residents undoubtedly translates into a better image for these places. That is, the residents’ perception of the territorial unit and opinion of it is more likely to be positive [19]. However, it should be remembered that the size, and condition, of the sports and recreation infrastructure primarily depends on the level of the country’s economic development and pro-health awareness of its citizens. Poland is significantly different from the European Union member countries in terms of the condition of its sports and recreation infrastructure (its number and quality, as well as the availability of specialist equipment). For example, the infrastructure in France is 6 times bigger than in Poland, in Sweden it is 5 times bigger, in Switzerland it is 10 times bigger, and in Hungary it is 13 times bigger. In this context, it is hardly surprising that the level of our society’s participation in physical culture is not high: It is estimated to be around 10% to 20% of the adult population [20]. For comparison, 59% of the population of Germany currently participates in physical activity, while in France 68% of the population is physically active and in the UK 65% of the population is physically active [21].

## 2. Aim of Study

Poznan is a city located in west-central Poland. The city’s population is about 540,000. It is an important academic site (with about 140,000 students), a center of trade, sports, technology, and tourism. It has often topped rankings as a city with very high-quality education and a very high standard of living [22]. It also ranks highly in safety and quality of healthcare [23].

The aim of the study is to identify which groups of factors (economic, social, and spatial) significantly determine the condition and development of the sports and recreation infrastructure of the city of Poznan and shape the needs and expectations of its residents.

## 3. Materials and Methods

### 3.1. Research Design and Data Collection

The literature on the subject shows that the development of sports and recreation infrastructure is determined, among other things, by economic, social, and spatial factors, which were compiled into three groups for the needs of the conducted works and the implementation of the research objective:

1. those that, according to the service recipients, create barriers in the use of sports and recreation facilities in the city of Poznan;
2. those which, in the opinion of service recipients, allow for the assessment of tasks contained in the “*Sportowy Poznań*” program;
3. those that, according to service providers, allow for the assessment of tasks contained in the “*Sportowy Poznań*” program.

Such a way of interpreting the research results allowed for the identification of the factors that, in the opinion of the surveyed service recipients and service providers, are the most important for the operation of sports and recreation facilities. It also allowed for the verification of the following thesis: Selected groups of factors significantly determine the condition and development of the sports and recreation infrastructure of the city of Poznan, as well as shape the needs and expectations of its residents. Simultaneously, the performed analysis constitutes the basis for the assessment of tasks contained in the “*Sportowy Poznań*” program.

The subjects of the research are indoor sports and recreation facilities (that offer the possibility to engage in sports and recreation regardless of the season) in Poznan. The questionnaire using standardized interviews made it possible to collect primary data concerning the presentation of the pace and directions of changes (trends) taking place in the sports and recreation facilities of the city of Poznan, paying special attention to identifying and prioritizing factors determining this development. Two self-constructed questionnaires were used for the study. These questionnaires were conducted by the authors among service providers and service recipients in the sports and recreation facilities in the areas of tennis courts, indoor swimming pools, and fitness clubs, and were personally filled out during a conversation with the recipients. The first part of the questionnaires focused on socio-demographic variables like age and education level (Table 1, Table 2 and Table 3). The second part of both questionnaires was strictly connected with the sports and recreation facilities in Poznan and was related to their availability and quality assessment. Service recipients were additionally divided into two groups: People professionally involved in sport and those who never practiced sport competitively. Both surveyed groups of respondents had the opportunity to mark the YES or NO answer in the questionnaire and to express their level of satisfaction using a specific point scale from 1 to 10 (1-unsatisfied, 10-highly unsatisfied).

### 3.2. Participants

A targeted selection of respondents (service providers and service recipients) was conducted in selected all-season sports and recreation facilities, and research was carried out in the areas of tennis courts (16 facilities, 480 respondents), indoor swimming pools (12 facilities, 360 respondents), and fitness clubs (11 facilities, 319 respondents) in the city of Poznan among 39 service providers (managing a sports and recreation facility) and 1159 service recipients (using the services of a given sports and recreation facility). The recipients were mainly 20–29 years old (49.3%, 572) and 30–39 years old (24.2%, 281). Among the surveyed people, the minority were aged 50 years old and older (3.6%, 42). A detailed specification is presented in Table 1, Table 2, and Table 3.

People with higher education constituted the vast majority of respondents at 55% (637). A total of 30.7% (356) possessed secondary education, and 14.3% (166) were people with primary education. The respondents who were professionally involved in sport comprised 41% (476), and those who never practiced sport competitively comprised 59% (683).

### 3.3. Data Analysis

To analyze the phenomena studied, the following methods of descriptive statistics were used: Arithmetic mean, measures of dispersion (variation, standard deviation), measures of the shape of the distribution (the value of skewness, kurtosis, tests for the equality of means ANOVA), tests for the equality of variances, Pearson’s chi-squared test, conditional probabilities, the binary variables model, and the Anderson–Darling Test. All statistical analyses were conducted using Statistica Software 10.0 (StatSoft Inc., Cracow, Poland, 2011).

The Anderson–Darling test is one of the statistical tests of distribution conformity with a set reference distribution model. It is usually used to check compliance with the normal distribution. It allows for the verification of hypotheses about the distribution of extreme statistics and characteristics of the variables studied in the work [24].

It is calculated as:

$$A^{2}=n\int_{-\infty }^{+\infty }\frac{\left(F_{n}\left(x\right)-F\left(x\right)\right)2}{F\left(x\right)\left(1-F\left(x\right)\right)}dF\left(x\right).$$

This test was used to establish the hierarchy of factors analyzed and studied in this work, operating within the same research problem (tasks of the “*Sportowy Poznań*” program) for both groups of respondents (service recipients and service providers).

## 4. Results

### 4.1. Factors Limiting the Residents’ Possibility of Using Sports and Recreation Facilities in the City of Poznan, Based on the Opinions of Service Recipients

The factors limiting the residents’ ability to use sports and recreation facilities in the city of Poznan based on the opinions of service recipients were put together in the following groups:

- Spatial (large distance of a sports and recreation facility from the place of residence, transport difficulties);
- Socio-spatial (general accessibility of the sports and recreation facility);
- Social (an object for competitive athletes only, no preferred sports and recreational activities);
- Economic (high fees in sports and recreation facilities).

Table 4 is a summary of the responses of the recipients in total, both amateurs and those competitively practicing sports. They indicate that, according to the respondents, the factors restricting their possibilities of using sports and recreation facilities (thus constituting barriers) have a similar impact on the level of satisfaction of the surveyed recipients, ranging between the mean values of $\bar{X}$ = 4.280 and $\bar{X}$ = 5.475.

In the opinion of the surveyed service recipients, the strongest limiting impact has an economic factor (high prices in sports and recreation facilities). This is indicated not only by the average value ($\bar{X}$ = 5.475), but also by the description of the shape of the distribution and the skewness coefficient contained in Table 1 (SK = −0.321), the result of which means that this factor was of great importance for the majority of surveyed service providers. Based on the opinions of the service recipients, the influence of other factors as limiting barriers is in the order: Spatial factors (long distance between a sports and recreational facility and the place of residence—$\bar{X}$ = 4.970) and lack of general accessibility of sports and recreation facilities—$\bar{X}$ = 4.764.

**Figure 1:**
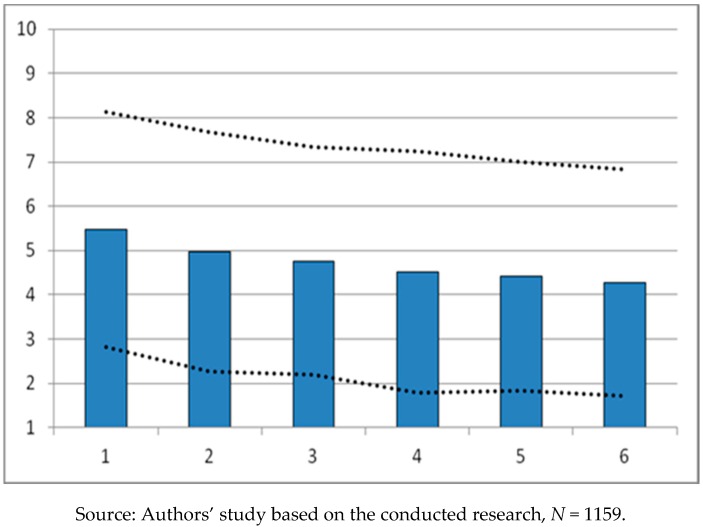
Barriers limiting the accessibility of sports and recreation facilities based on the opinions of service recipients—$\bar{X}$ ± S. Figure Legend: 1. High prices in a sports and recreation facility; 2. Long distance between a sports and recreational facility and the place of residence; 3. General accessibility of a sports and recreation facility; 4. Lack of preferred activities in a sports and recreation facility; 5. Transport difficulties; 6. The object is intended for competitive athletes.

The graphic in Figure 1 shows the scale of the impact of the studied factors on the limitation of the accessibility of sports and recreation facilities. The least significant barrier, according to the surveyed service recipients, is a social factor, indicating that a sports and recreation facility is mainly intended for competitive athletes (average level of satisfaction of respondents—$\bar{X}$ = 4.280). Despite the small gradation existing in the importance of the selected factors, it is worth emphasizing that in the opinion of the surveyed service recipients, all of these factors matter and all of them cause more or less significant restrictions on the use of sports and recreation facilities in the city of Poznan.

### 4.2. The Importance of Individual Factors in the Implementation of the “*Sportowy Poznań*” Strategic Program, Based on the Opinions of Service Recipients

The next step in the adopted research procedure was an attempt to assess the significance of action/performance factors resulting from tasks included in the strategic program for the city of Poznan, “*Sportowy Poznań*” The assessment is based on the opinions of both the surveyed service recipients (Table 5) and service providers (Table 6) concerning the tasks included in the program. The factors assessed were compiled into the following groups:

- social (preparing sports and recreation infrastructure for the residents of the city of Poznan, propagating the idea of a healthy and active lifestyle among the residents of the city of Poznan, promoting and organizing sport for children and teenagers, supporting the physical activity of residents through mass sport);
- socio-economic (professionalism of the operation of sports clubs in Poznan, creating the image of the city of Poznan through sports and recreation, Poznan as an international center of sports and recreation);
- spatial-economic (extension of sports and recreation infrastructure).

Table 5 contains an assessment of the importance of the individual performance factors of the “*Sportowy Poznań*” strategic program based on the opinions of the recipients.

**Figure 2:**
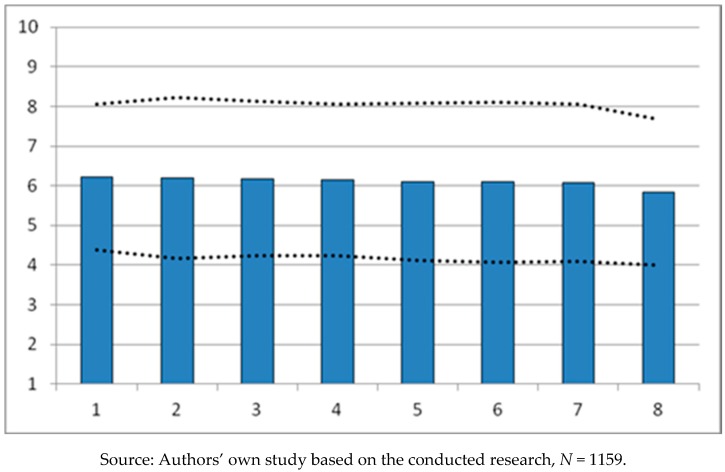
Graphic image of arithmetic means $\bar{X}$ ± S of individual factors, based on the opinions of service recipients. Figure Legend: 1. Preparation of infrastructure for residents; 2. Promoting the idea of an active lifestyle; 3. Creating the city’s image through sports and recreation; 4. Expansion of infrastructure; 5. Poznan as an international sports center; 6. Supporting mass sport; 7. Promoting sports for children and teenagers; 8. Operation of sports clubs.

The level of satisfaction of the surveyed service recipients, illustrated by the average values contained in Table 5, is their opinion on the effectiveness of the policy conducted by the City of Poznan regarding activities aimed at enriching the sports and recreation offer for residents, promoting an active and healthy lifestyle among residents and increasing the significance of the city of Poznan as a center of sports and recreation. The responding service recipients quite highly assessed the efforts of the authorities in the implementation of particular tasks of the “*Sportowy Poznań*” program. Almost all of the factors examined in the work were considered significant, obtaining the average values of $\bar{X}$ = 6.223 to $\bar{X}$ = 6.076. It is worth emphasizing that the values illustrating the level of satisfaction of the surveyed service recipients are very similar to each other in the assessment of individual factors. Figure 2 is a hierarchical comparison of the significance of the factors studied. The respondents granted the greatest importance and satisfaction with the effects of implementation to the social factor of the preparation of sports and recreation infrastructure for the residents of the city of Poznan. The importance of other factors as part of the implementation of the “*Sportowy Poznań*” program by the City of Poznan authorities is similar and is at a similar level. Only the functioning, or rather the efforts of the authorities to operate Poznan sports clubs in a professional manner, gained the lowest score in the opinion of the surveyed service recipients ($\bar{X}$ = 5.832). Summarizing the results of the research contained in Table 5 and illustrated in Figure 2, it can be stated that the surveyed service recipients positively assessed the effects of the implementation of the “*Sportowy Poznań*” program, thus granting similarly high significance to the factors distinguished in the work. The conformity of opinions among service recipients is illustrated by the values of the means.

### 4.3. The Importance of Individual Factors in the Implementation of the “*Sportowy Poznań*” Strategic Program, Based on the Opinions of Service Providers

**Figure 3:**
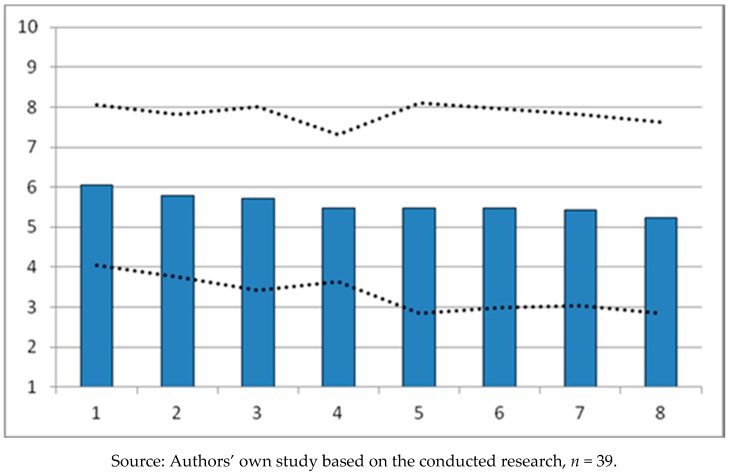
Graphic image of arithmetic means $\bar{X}$ ± S of individual factors, based on the opinions of service providers. Figure Legend: 1. Preparation of infrastructure for residents; 2. Expansion of infrastructure; 3. Promoting the idea of an active lifestyle; 4. Promoting sports for children and teenagers; 5. Poznan as an international sports center; 6. Supporting mass sport; 7. Creating the city’s image through sports and recreation; 8. Operation of sports clubs.

The implementation of individual tasks in the “*Sportowy Poznań*” program was also evaluated by the surveyed service providers. The results, expressing the opinion that the importance of particular performance factors of the “*Sportowy Poznań*” program, are included in Table 6 and Figure 3.

The effectiveness of the city authorities’ policy in implementing the “*Sportowy Poznań*” program has been assessed by the surveyed service providers as average. The importance of individual factors emerging in the work, based on the opinions of the service providers, is similar and is expressed in average values ranging from $\bar{X}$ = 6.053 to $\bar{X}$ = 5.237 (Table 6). The values of the means that determine the level of satisfaction of the respondents are similar to each other, but lower than the mean values obtained in the assessment made by the recipients. The surveyed service providers recognize the significant impact of all of the identified factors, and like the service recipients, they distinguish the social factor of the preparation of infrastructure for residents of Poznan with a higher average ($\bar{X}$ = 6.053), thus granting it a greater significance in the effects of the implementation. According to the surveyed service providers, another factor of great importance is the spatial factor of the development of sports and recreation infrastructure ($\bar{X}$ = 5.789). In this way, the surveyed service providers acknowledged that the actions of the authorities related to the appropriate preparation and development of sports and recreation infrastructure as part of the tasks covered by the “*Sportowy Poznań*” program have all been implemented in a satisfactory manner. The service providers gave the lowest marks to the effects of activities regarding the professionalism of Poznan sports clubs ($\bar{X}$ = 5.237). Thus, they confirmed the opinion expressed earlier by the surveyed service recipients. Figure 3 is a hierarchical comparison of the importance of individual factors in the implementation of the “*Sportowy Poznań*” program based on the opinions of service providers. The surveyed service providers positively assessed the effects of the implementation of the “*Sportowy Poznań*” program, awarding similar significance to the factors distinguished in the work. It is worth noting, however, that in the opinion of the service providers, the level of operation of these factors is lower than that expressed by the service recipients. This is worth emphasizing due to the fact that the service providers objectively assessed the functioning of the sports and recreation facility they run, without trying to artificially raise its rank.

The research procedure was designed to identify factors that were considered equally important by both groups of respondents. Such indications can and should be a basis for city authorities’ creation of effective policy for the development of sports and recreation infrastructure, thus affecting the recreational activity of the residents of the city of Poznan. In order to establish the hierarchy of factors analyzed in the paper and operating within the same research problem for both groups of respondents (service recipients and service providers), the Anderson-Darling test was used. This test allows for the verification of hypotheses about the distribution of extreme statistics, characteristic of the variables studied in the work. The adopted hypothesis assumes that the probability distribution obtained for service recipients is identical to the probability distribution obtained for service providers. The test results contained in Table 7 clearly indicate that the social factor, i.e., the preparation of infrastructure for the residents of the city of Poznan, was considered the most important in the opinion of both surveyed groups (service recipients and service providers), obtaining a test value of 0.886. The remaining factors discussed in the work obtained much lower values with this test in relation to the above-mentioned factor. The Anderson–Darling test placed a spatial and economic factor regarding the development of sports and recreation infrastructure second in the hierarchy, with a test value of 0.362. The third one, with a test value of 0.207, close to the previous one, is another social factor in the hierarchy, propagating the idea of an active lifestyle for the inhabitants of Poznan. The lowest importance in the hierarchy of factors, with a test value of 0.017, was the socio-economic factor of creating the image of the city of Poznan through sports and recreation.

The application of the Anderson–Darling test allowed for a clear identification of the determinant with the highest impact on the development of the sports and recreation infrastructure in the city of Poznan, thus enabling the residents to participate in recreation. It should be emphasized that both groups of respondents jointly regarded this factor as the most important, which means that its implementation, consisting of the preparation of infrastructure for the residents of the city of Poznan, meets the expectations of both users and managers of sports and recreation facilities. The negative result of the socio-economic factor of creating the image of the city of Poznan through sports and recreation is equally important in the test. It means that the activities of the City of Poznan authorities in this respect do not meet the expectations of the city’s residents.

The data quoted below contain the opinions of Poznan residents surveyed against the background of surveys conducted by the Central Statistical Office, which present the opinions of respondents from Poland and other European Union citizens. The comparative analysis shows the following:

- Environment and possibilities of being active: In Poznan, the level of satisfaction of the residents surveyed was average, expressed as $\bar{X}$, around 5 on a scale from 1 to 10.
- The offer of local sports clubs and other service providers (swimming pools, fitness clubs, etc.): In Poznan, the level of satisfaction of the surveyed residents is above the average, expressed as $\bar{X}$, about a 7 on a scale from 1 to 10.
- Evaluation of the local authorities’ policies aimed at improving the physical activity of residents: In Poznan, the level of satisfaction of the surveyed residents ranks above the average expressed in the value $\bar{X}$, above a 6 on a scale from 1 to 10.

## 5. Discussion and Final Conclusions

In the majority of European Union countries, citizens are satisfied with the efforts of local authorities to ensure that local residents have the possibility to engage in sports and recreation activities [14], but Polish respondents believe that local authorities do not do enough in this area. This was partially confirmed by the opinion of the Poznan residents surveyed in this work. Among the European Union guidelines on physical activity, the most important are: Access to sports and recreation infrastructure and, what is important, free or inexpensive use of public sports and leisure facilities [25]. Such premises should be guided by the city of Poznan authorities by engaging in the development of sports and recreation facilities, supporting local clubs and promoting a healthy, active life of residents. This was confirmed by the results of the research carried out in this work, where the respondents in Poznan and the service providers agreed that the most important in the implementation of the City Hall’s policy was the appropriate development of sports and recreation infrastructure along with its expansion and promotion of the active lifestyle of residents.

The aim of the article is to identify which groups of factors (economic, social, and spatial) significantly determine the condition and development of the sports and recreation infrastructure of the city of Poznan and shape the needs and expectations of its residents. For the needs of the conducted work and implementation of the research objective, the determinants are divided into three groups:

1. those that, according to service recipients, create barriers for the use of sports and recreation facilities in the city of Poznan;
2. those which, in the opinion of service recipients, allow for the assessment of tasks contained in the “*Sportowy Poznań*” program;
3. those that, according to service providers, allow for the assessment of tasks contained in the “*Sportowy Poznań*” program.

The subjects of the research are indoor sports and recreation facilities in Poznan (that provide the possibility to engage in sports and recreation regardless of the season). Standardized interviews allowed for the collection of primary data among service recipients (*N* = 1159) and service providers (*n* = 39) regarding the presentation of pace and development trends in sports and recreation facilities in the city of Poznan, paying particular attention to identifying and prioritizing factors determining this development. The conducted research procedure allowed for the verification of the assumptions and objectives pursued and permitted in the following conclusions:

1. Among the accepted factors of the operation of the “*Sportowy Poznań*” strategic program, based on the opinions of service recipients, the economic factor has the strongest limiting effect on the use of sports and recreation services (high prices in sports and recreation facilities). Then there are spatial factors (long distance between the sports and recreation facility and the place of residence) and the lack of general accessibility of sports and recreation facilities.
2. In the implementation of the “*Sportowy Poznań*” strategic program, the surveyed service recipients granted the greatest importance and satisfaction with the effects of the implementation to the social factor—preparation of sports and recreation infrastructure for the residents of the city of Poznan. Efforts of the city authorities to ensure the professional operation of Poznan sports clubs gained the lowest score in the opinion of the surveyed service recipients. The activity of sports clubs to a large extent creates the image of the city as a center for sports.
3. The effectiveness of the city authorities’ policies in the implementation of the “*Sportowy Poznań*” strategic program was assessed as average by service providers. The values of the means determining the level of satisfaction of the respondents are comparable, but lower than the average values obtained in the assessment made by the service recipients. Service providers objectively assessed the functioning of the sports and recreation facility they run, without trying to artificially raise its rank. The surveyed service providers recognize the significant impact of social and spatial factors, judging them as satisfactory, as they do with all of the tasks covered in the “*Sportowy Poznań*” program. The lowest score was given by service providers (as well as service recipients) to the effects of activities related to the professionalism of the operation of Poznan sports clubs.
4. In order to establish the hierarchy of factors analyzed in the paper and operating within the same research problem for both groups of respondents (service recipients and service providers), the Anderson–Darling test was used. The test results (Table 7) clearly indicate that:
  - The social factor of the preparation of infrastructure for the residents of the city of Poznan, in the opinion of both groups of respondents (service recipients and service providers), was considered the most important, obtaining a test value of 0.886;
  - A spatial-economic factor came second in the hierarchy of the Anderson–Darling test, with a test value of 0.362;
  - The third in the hierarchy (with a test value similar to the previous 0.207) was a social factor propagating the idea of an active lifestyle for the residents of the city of Poznan;
  - The lowest importance in the hierarchy of factors, with a test value of 0.017, was achieved by the socio-economic factor of creating the image of the city of Poznan through sports and recreation.

It should be emphasized that the social factor based on the preparation of infrastructure for the residents of the city of Poznan was jointly selected as the most important by both groups of respondents, which means that its implementation, consisting of preparing infrastructure for the residents of Poznan, meets the expectations of both users and managers of sports and recreation facilities. The negative result of the socio-economic factor of creating the image of the city of Poznan through sports and recreation obtained in the test is equally important. It means that the activities of the city of Poznan authorities in this respect do not meet the expectations of the city’s residents. Unfortunately, in the opinion of the surveyed service recipients, the economic factor turned out to be the one that most severely limited the availability of sports and recreation facilities in the city of Poznan. The results of the research show that the inhabitants of Poznan understand how important it is for them to provide conditions for the implementation of various forms of physical activity by building facilities for sports and recreation accessible, and providing them with adequate equipment, that is, the preparation of sports and recreation infrastructure in the city. While analyzing the responses of the surveyed service recipients, attention should also be paid to their opinion concerning supporting mass sports in Poznan, as this is one of the most significant factors determining the physical activity of the city’s residents. If sport for all does not find much support from city residents, it may result from the lack of a rational development strategy not only in terms of infrastructure, but also in terms of staff.

## Figures and Tables

**Table 1:** Characteristics of respondents.

Age Characteristics of the Respondents	Tennis Courts (*n* = 480)		Indoor Swimming Pools (*n* = 360)		Fitness Clubs (*n* = 319)		All (*N* = 1159)
	*n*	%	*n*	%	*n*	%	
<20	78	17.3	16	7.8	92	17.5	186
20–29	225	49.8	99	48.5	248	47.1	572
30–39	96	21.2	67	32.8	118	22.4	281
40–49	35	7.7	13	6.4	54	10.2	102
50+	18	4.0	9	4.4	15	2.8	42

**Table 2:** Descriptive statistics—for observations from sample *N* = 1159 for the age variable.

Mean	Median	Minimum	Maximum
27.7990	25.0000	15.0000	67.0000
Standard Deviation	Coefficient of Variation	Skewness	Kurtosis
9.66253	0.347586	1.20478	1.52895

**Table 3:** Descriptive statistics—for observations from sample *N* = 1159 for the age variable in each object.

	Mean	Median	Minimum	Maximum
Indoor swimming pools	27.9624	25.0000	15.0000	67.0000
Tennis courts	29.3676	27.0000	15.0000	60.0000
Fitness clubs	27.7780	25.0000	16.0000	56.0000
	Standard Deviation	Coefficient of Variation	Skewness	Kurtosis
Indoor swimming pools	10.4107	0.372310	1.42351	2.25329
Tennis courts	8.93860	0.304369	0.806148	0.216468
Fitness clubs	9.08693	0.327127	0.829227	−0.00652774

**Table 4:** Statistical characteristics of factors restricting the possibility of using sports and recreation facilities, based on the opinions of service recipients.

Specification of Factors	$\bar{X}$ Arithmetic Mean	S Standard Deviation	SK Skewness Coefficient	K Kurtosis	All (*N* = 1159)
General accessibility—socio-spatial factor	4.764	2.664	0.055	2.254	1159
Long distance—spatial factor	4.970	2.692	‒0.092	2.160	1159
Transport problems/difficulties—spatial factor	4.422	2.563	0.128	2.256	1159
High prices—economic factor	5.475	2.736	321	2.362	1159
Object designed for competitive athletes—social factor	4.280	2.585	0.248	2.487	1159
Lack of preferred activities—social factor	4.513	2.565	‒0.010	2.313	1159

Source: Authors’ own study based on the conducted research, *N* = 1159.

**Table 5:** Statistical characteristics of individual factors based on the opinions of service recipients.

“Sportowy Poznań”	$\bar{X}$ Arithmetic Mean	S Standard Deviation	SK Skewness Coefficient	K Kurtosis	All (*N* = 1159)
Preparation of infrastructure for residents—social factor	6.223	1.835	296	3.113	1159
Promoting the idea of an active lifestyle—social factor	6.200	2.031	258	2.724	1159
Promoting sports for children and teenagers—social factor	6.076	1.988	‒0.297	2.901	1159
Expansion of infrastructure—spatial and economic factor	6.145	1.912	‒0.244	2.856	1159
Poznan as an international sports center—socio-economic factor	6.101	1.972	‒0.362	2.878	1159
Supporting mass sport—social factor	6.088	2.013	‒0.173	2.628	1159
Operation of sports clubs—socio-economic factor	5.832	1.832	‒0.101	2.857	1159
Creating the city’s image through sports and recreation—socio-economic factor	6.180	1.943	‒0.401	2.959	1159

Source: Authors’ own study based on the conducted research, *N* = 1159.

**Table 6:** Statistical characteristics of the importance of individual factors based on the opinions of service providers.

“Sportowy Poznań”	$\bar{X}$ Arithmetic Mean	S Standard Deviation	SK Skewness Coefficient	K Kurtosis	All (*N* = 1159)
Preparation of infrastructure for residents—social factor	6.053	2.013	−0.46	3.687	38
Promoting the idea of an active lifestyle—social factor	5.711	2.301	−0.49	3.262	38
Promoting sports for children and teenagers —social factor	5.474	1.842	−0.85	4.189	38
Expansion of infrastructure—spatial and economic factor	5.789	2.029	−0.56	4.139	38
Poznan as an international sports center—socio-economic factor	5.747	2.638	−0.48	2.73	38
Supporting mass sport—social factor	5.474	2.480	−0.40	3.133	38
Operation of sports clubs—socio-economic factor	5.237	2.399	−0.24	3.314	38
Creating the city’s image through sports and recreation—socio-economic factor	5.421	2.390	−0.55	3.417	38

Source: Authors’ own study based on the conducted research, *n* = 39.

**Table 7:** Values of the Anderson–Darling test—hierarchy of factors determining the development of sports and recreation infrastructure.

Tasks of the “*Sportowy Poznań*” Program	Factors	Anderson–Darling Test *p*-Value
Preparation of infrastructure for residents	Social factor	0.886
Promoting the idea of an active lifestyle	Social factor	0.207
Promoting sports for children and teenagers	Social factor	0.046
Expansion of infrastructure	Spatial and economic factor	0.362
Poznan as an international sports center	Socio-economic factor	0.045
Supporting mass sport	Social factor	0.069
Operation of sports clubs	Socio-economic factor	0.031
Creating the city’s image through sports and recreation	Socio-economic factor	0.017

Source: Authors’ own study based on the conducted research.
